# Fetal and Neonatal Nicotine Exposure in Wistar Rats Causes Progressive Pancreatic Mitochondrial Damage and Beta Cell Dysfunction

**DOI:** 10.1371/journal.pone.0003371

**Published:** 2008-10-08

**Authors:** Jennifer E. Bruin, Maria A. Petre, Sandeep Raha, Katherine M. Morrison, Hertzel C. Gerstein, Alison C. Holloway

**Affiliations:** 1 Reproductive Biology Division, Department of Obstetrics and Gynecology, McMaster University, Hamilton, Ontario, Canada; 2 Department of Pediatrics, McMaster University, Hamilton, Ontario, Canada; 3 Department of Medicine, McMaster University, Hamilton, Ontario, Canada; Mayo Clinic College of Medicine, United States of America

## Abstract

Nicotine replacement therapy (NRT) is currently recommended as a safe smoking cessation aid for pregnant women. However, fetal and neonatal nicotine exposure in rats causes mitochondrial-mediated beta cell apoptosis at weaning, and adult-onset dysglycemia, which we hypothesize is related to progressive mitochondrial dysfunction in the pancreas. Therefore in this study we examined the effect of fetal and neonatal exposure to nicotine on pancreatic mitochondrial structure and function during postnatal development. Female Wistar rats were given saline (vehicle control) or nicotine bitartrate (1 mg/kg/d) via subcutaneous injection for 2 weeks prior to mating until weaning. At 3–4, 15 and 26 weeks of age, oral glucose tolerance tests were performed, and pancreas tissue was collected for electron microscopy, enzyme activity assays and islet isolation. Following nicotine exposure mitochondrial structural abnormalities were observed beginning at 3 weeks and worsened with advancing age. Importantly the appearance of these structural defects in nicotine-exposed animals preceded the onset of glucose intolerance. Nicotine exposure also resulted in significantly reduced pancreatic respiratory chain enzyme activity, degranulation of beta cells, elevated islet oxidative stress and impaired glucose-stimulated insulin secretion compared to saline controls at 26 weeks of age. Taken together, these data suggest that maternal nicotine use during pregnancy results in postnatal mitochondrial dysfunction that may explain, in part, the dysglycemia observed in the offspring from this animal model. These results clearly indicate that further investigation into the safety of NRT use during pregnancy is warranted.

## Introduction

Cigarette smoking is associated with numerous adverse obstetrical and fetal outcomes [1]–[6], yet 15–20% of women reportedly smoke during pregnancy [1], [7]. Furthermore, mounting epidemiologic evidence indicates that maternal smoking is associated with an increased risk of obesity, hypertension and type 2 diabetes in the offspring [7]–[13], although the mechanisms underlying this relationship are unknown. Our laboratory has previously demonstrated in a rat model that maternal exposure to nicotine, the major addictive component of cigarettes, during pregnancy and lactation results in postnatal obesity and impaired glucose homeostasis in adult offspring [14], [15]. Because nicotine replacement therapy (NRT) is recommended for pregnant women who cannot quit smoking by other means [16], these results may have significant public health implications. In our animal model, postnatal dysglycemia following fetal and neonatal nicotine exposure was associated with a loss of beta cell mass, beginning at birth and persisting into adulthood [14]. This reduction in beta cell mass following developmental nicotine exposure may partially explain the increased risk of type 2 diabetes in the offspring of women who smoked during pregnancy [8].

Individuals with type 2 diabetes are unable to produce sufficient insulin to maintain normal glucose homeostasis [17]. This has been attributed, in part, to reduced beta cell mass and impaired beta cell function [17], [18]. In beta cells, the mitochondria are involved in triggering apoptosis, thereby contributing to the regulation of beta cell mass [19], [20]. We have previously shown that fetal and neonatal exposure to nicotine results in beta cell loss due to increased oxidative stress [21] and beta cell apoptosis [14], [15]. Furthermore, we have demonstrated that this nicotine-induced oxidative stress differentially targeted the mitochondria in the pancreas [21], resulting in mitochondrial-mediated beta cell apoptosis [22]. However, in addition to regulating beta cell mass (via apoptosis), the mitochondria are also critical for maintenance of beta cell function through the coupling of a glucose stimulus to insulin release [23]–[25]. Both human and animal studies have demonstrated mitochondrial dysfunction in islets of subjects with type 2 diabetes [26], [27]. Therefore, we hypothesize that the dysglycemia observed in this animal model following fetal and neonatal nicotine exposure is likely mediated by pancreatic mitochondrial defects. This study will examine the effect of fetal and neonatal exposure to nicotine on postnatal mitochondrial structure and function, as well as subsequent beta cell function.

## Methods

### Maintenance and treatment of animals

All animal experiments were approved by the Animal Research Ethics Board at McMaster University, in accordance with the guidelines of the Canadian Council for Animal Care. Nulliparous 200–250 g female Wistar rats (Harlan, Indianapolis, IN, USA) were maintained under controlled lighting (12∶12 L∶D) and temperature (22°C) with *ad libitum* access to food and water. Dams were randomly assigned (n = 30 per group) to receive saline (vehicle) or nicotine bitartrate (1 mg·kg^−1^·d^−1^, Sigma-Aldrich, St. Louis, MO, USA) via subcutaneous injection daily for 2 weeks prior to mating until weaning (postnatal day 21). We have previously demonstrated that this dose of nicotine (1 mg·kg^−1^·d^−1^) results in cotinine concentrations in maternal serum that are similar to “moderate” female smokers and in nicotine-exposed offspring serum at birth that are comparable to infants nursed by smoking mothers [28]. At postnatal day 1, litters were culled to eight to assure uniformity of litter size between treated and control litters. To eliminate any confounding effects of the female reproductive cycle, only male offspring were used in this study.

### Oral glucose tolerance

Glucose homeostasis was investigated in nicotine-exposed and saline control rats at 4, 15 and 26 weeks of age (n = 15 per group) using sequential oral glucose tolerance tests (OGTT) as previously described [14], [15]. Briefly, after an overnight fast insulin and glucose were measured in saphenous vein samples, collected by repeated puncture, at baseline, 30 and 120 minutes after rats were given 2 g·kg^−1^ glucose (Sigma-Aldrich, St. Louis, MO, USA) in water by gavage. Blood samples were allowed to clot at 4°C, centrifuged and stored at −80°C until assayed. Serum glucose concentrations were measured by a commercially available kit using the glucose oxidase method (Pointe Scientific Inc., Canton, MI, USA), and insulin levels were measured by an ultra sensitive rat insulin ELISA (Crystal Chem Inc., Downers Grove, IL, USA). Data are presented as the average area under the curve (AUC)±SEM for saline- and nicotine-exposed offspring at each age.

### Electron microscopy

Pancreas tissue from offspring at 3 weeks (n = 4 per group), 15 weeks (saline: n = 3 and nicotine: n = 4), and 26 weeks (n = 3 per group) were collected and processed for electron microscopy as previously described [22]. All chemicals used for electron microscopy were purchased from Canemco Inc., Montreal, QC, Canada unless otherwise stated. Thick sections (approximately 1 µm) were cut on an Ultracut E ultramicrotome (Leica Microsystems, Wetzlar, Germany), stained with toluidine blue and examined under a light microscope to ensure the presence of islets. Thin sections (approximately 70 nm) were then cut from areas of the tissue containing islets, mounted on a Cu/Pd grid (200 mesh), and stained with saturated uranyl acetate and lead citrate. Grids were examined with a JEOL 1200EX transmission electron microscope (JEOL Ltd., Tokyo, Japan) and representative photographs were taken at either 5000× or 12000× magnification. All photographs were analysed by a single investigator blinded to the treatment groups using Image Pro Plus Version 5.1 software (Media Cybernetics, Inc., Silver Spring, MD, USA).

**Figure 1:**
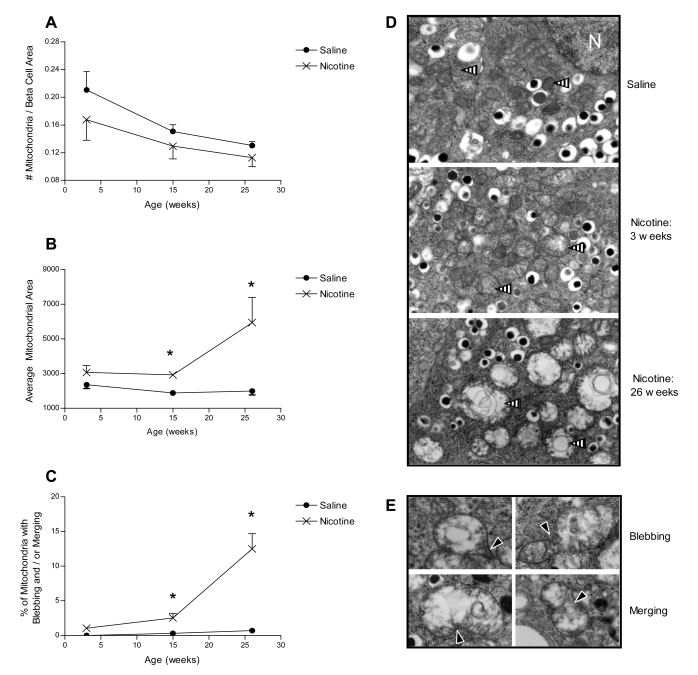
Mitochondrial morphology during postnatal development. A) The number of mitochondria per beta cell area; B) individual mitochondrion area; and C) percentage of mitochondria with blebbing and/or merging with a neighboring mitochondria from offspring at 3, 15 and 26 weeks of age following exposure to either saline or nicotine during fetal and neonatal development. Representative electron microscopy photographs are provided to illustrate: D) typical mitochondrial structure (indicated by striped arrows) in the beta cells of saline and nicotine-exposed offspring during postnatal development, and E) examples of mitochondrial blebbing and merging (indicated by solid black arrows); N = nucleus. All data are presented as the mean±SEM. Values with an asterisk are significantly different from the saline control (p<0.05).

**Figure 2:**
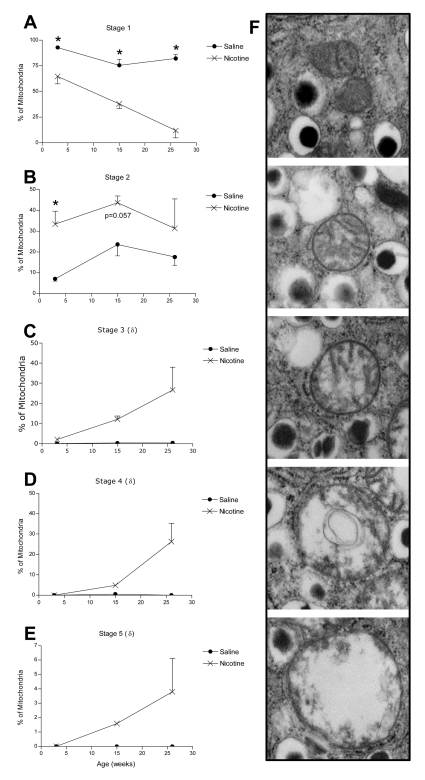
Stages of mitochondrial health during postnatal development. The percentage of mitochondria within beta cells at: A) stage 1, B) stage 2, C) stage 3, D) stage 4, and E) stage 5. F) Electron microscopy images with examples of mitochondria at each of the five morphological stages. δ indicates that statistical analysis was not performed on these data due to lack of variability in the saline treatment group replicates. All data are presented as mean±SEM.

Beta cells were identified within the pancreas sections by the presence of insulin granules. Insulin granules were classified as filled (dense-core), immature (light gray granule) or empty (no insulin). The number of insulin granules and mitochondria were calculated relative to the area of a beta cell. Individual mitochondrial morphology was assessed by quantifying: a) the average mitochondrion area; b) the proportion of mitochondria with blebbing and/or merging with other mitochondria (refer to Figure 1E for examples); c) the proportion of mitochondria in each of five defined stages of progressive deterioration. The definitions for each mitochondrial stage were created using a modification of a previously described scale for assessing mitochondrial morphologies [29]. Stage 1 mitochondria were classified as structurally healthy, with dense, intact cristae. Stage 2 mitochondria had visible swelling, but maintained distinctive intact cristae structure. Stage 3 mitochondria had more severe swelling and minimal evidence of intact cristae. Stage 4 mitochondria displayed severe swelling, minimal cristae structure and formation of vacuoles. Stage 5 mitochondria were extremely large and swollen, with essentially complete loss of defined structure within the mitochondrial membrane. An example of mitochondria at each defined morphological stage is provided in Figure 2F.

### Mitochondrial enzyme activity

Pancreas tissue was excised from offspring at 3 weeks (saline: n = 7, nicotine: n = 5), 15 weeks (n = 5 per group) and 26 weeks (n = 5 per group), frozen on dry ice and stored at −80°C until analysis. Tissue samples were homogenized in homogenization buffer (5 mM HEPES pH 7.4, 100 mM KCl, 70 mM sucrose, 220 mM mannitol, 1 mM EGTA) with Complete Mini EDTA-free protease inhibitors (Roche Applied Science, Laval, QC, Canada) using Tenbroeck tissue grinders. Homogenates were spun for 10 min at 600×g, the supernatant removed, flash frozen in liquid nitrogen and stored at −80°C until use. Citrate synthase activity (an indicator of total mitochondrial mass) was measured using the thiol reagent 5,5′-dithio-bis-(2-nitrobenzoic acid) (DTNB, Sigma Chemical Co., St. Louis, MO, USA). Complex IV (cytochrome *c* oxidase) activity was assessed by measuring the rate of cytochrome *c* (from equine heart; Sigma Chemical Co., St. Louis, MO, USA) oxidation. Both activity assays were performed using UV-spectrophotometry (Varian Inc., Palo Alto, CA, USA) as previously described [30]. Data are expressed as the mean enzyme activity relative to the wet weight of tissue.

### Islet isolation

Islet isolation was performed as previously described [21] at 26 weeks of age. Briefly, the pancreas was immediately excised following sacrifice, minced finely and placed in 6 mL of Hank's balanced salt solution (HBSS) (HyClone, Logan, UT, USA) containing 4 mg/mL collagenase type IA (Sigma-Aldrich, St. Louis, MO, USA), 100 IU/mL penicillin G and 0.25 µg/mL streptomycin (Gibco, Grand Island, NY, USA). Following an incubation period of 40 min at 37°C, the reaction was quenched with 20 mL HBSS supplemented with 10% fetal bovine serum (FBS) (HyClone, Logan, UT, USA), 100 IU/mL penicillin G and 0.25 µg/mL streptomycin. Islets were manually picked from the suspension using a small glass pipette and a dissecting microscope. The islets were incubated at 37°C, 5% CO_2_ /95% normal atmosphere in 5 mL RPMI 1640 with 3.0 mM glucose (Life Technologies, Burlington, ON, Canada) supplemented with 10% FBS, 100 IU penicillin G and 0.25 µg/mL streptomycin for 24 hours.

### Reactive oxygen species production by isolated islets

Reactive oxygen species (ROS) production by isolated islets following saline and nicotine exposure was measured using 2′,7′-dichlorodihydrofluorescein diacetate (H_2_DCFDA) (Molecular Probes Inc., Eugene, OR, USA) fluorescence as previously described [21]. Since islet ROS production at weaning has been previously confirmed in this animal model [21], oxidative stress was only assessed at the endpoint of the current study (26 weeks). Briefly, 80 islets from saline- and nicotine-exposed offspring (n = 6 per group) were washed with PBS. Following centrifugation, the supernatant was removed and the pelleted islets were then resuspended in 100 µL of PBS containing 100 µM H_2_DCFDA and incubated for 3 h at 37°C. Because of the relatively low number of cells in this assay, a long incubation period allows for the diffusion of the oxidized dye from inside the cell back out into the culture medium [27]. This approach has been previously validated to determine ROS production by isolated islet cells in rats [21], [27]. In addition, since H_2_DCFDA must be made fresh immediately prior to use, islets isolated on different days were incubated in different batches of reagent. To account for day-to-day variability within the experiment, a 43 µM hydrogen peroxide reaction was prepared with each batch of H_2_DCFDA to calibrate the performance of the dye. The hydrogen peroxide was added to 100 µM H_2_DCFDA and incubated in parallel with the islet reactions. Following the incubation period, the islets were vigorously disrupted to release intracellular H_2_DCFDA. Both the islet suspensions and the hydrogen peroxide control were centrifuged, and the supernatants were transferred to black 96-well plates (BD Falcon, Mississauga, ON, Canada). Fluorescence of the 2′,7′-dichlorofluorescein product was determined using a SpectaMax Gemini XS (Molecular Devices Corp., Sunnyvale, CA, USA) microplate spectrofluorometer at excitation and emission wavelengths of 505 nm and 540 nm, respectively. All measurements of islet ROS production were normalized to the 43 µM hydrogen peroxide control and expressed as a percentage of the average saline control.

### Oxyblot detection of protein carbonyls in isolated islets

To assess oxidative damage by reactive oxygen species to islet proteins, the presence of protein carbonyl groups was quantified using the OxyBlot^TM^ Protein Oxidation Detection Kit (Chemicon International, Temecula, CA, USA). Formation of protein carbonyl groups was measured at 26 weeks in isolated pancreatic islets (saline: n = 6, nicotine: n = 5). 100 islets were hand-picked into eppendorf tubes and centrifuged for 3 minutes at 300 rcf. The supernatant was removed and islets were resuspended in 100 µL of homogenization buffer with protease inhibitors (as described above) and frozen at −80°C until use. Upon thawing, cells were lysed using a sonication probe. Protein samples (5 µL) were then prepared with the Oxyblot^TM^ Kit, according to manufacturer's instructions. Derivatized protein was subjected to SDS-PAGE using a 12 % separating gel and then electro-transferred to PVDF blotting membrane (BioRad Laboratories, Hercules, CA, USA). Membranes were blocked for 2 h at room temperature with 5 % (w/v) skim milk in TBST (TBS, 0.5% (v/v) Tween 20), incubated overnight at 4°C in rabbit-DNP antibody (1∶150), and finally 1 h at room temperature in secondary goat anti-rabbit IgG (HRP-conjugated; 1∶300). Blots were washed thoroughly in TBST followed by TBS after immunoblotting. Reactive protein was detected with ECL Plus chemiluminescence (Amersham Biosciences, Piscataway, NJ, USA) and Bioflex X-ray film (Clonex Corporation, Markham, ON, Canada). Densitometric analysis of immunoblots was performed using ImageJ 1.37 v software (National Institutes of Health, Bethesda, MD, USA); all proteins were quantified relative to a Ponceau S (Sigma Aldrich, St. Louis, MO, USA) loading control.

### Glucose-stimulated insulin secretion in isolated islets

Glucose stimulated insulin secretion (GSIS) was examined as a marker of beta cell function at 26 weeks of age. Briefly, 20 islets from both saline- and nicotine-exposed offspring (n = 6 per group) were incubated in 100 µL of Krebs Ringer Bicarbonate buffer, pH 7.4 (135 mM NaCl, 3.6 mM KCl, 5 mM NaHCO_3_, 0.5 mM NaH_2_PO_4_·2H_2_O, 0.5 mM MgCl_2_·6H_2_O, 1.5 mM CaCl_2_·2H_2_O, 10 mM Hepes, 0.1% BSA) with either 3.0 mM glucose (basal) or 16.7 mM glucose (stimulated) for 2 hours at 37°C. All reactions were performed in duplicate. Following the incubation, islets were centrifuged, the media removed and stored at −80°C until use. The pelleted islets were resuspended in 25 µL of cell homogenization buffer (as described above), sonicated to release insulin and frozen at −80°C until use. Insulin levels were measured in both the media and the pellet by an ultra sensitive rat insulin ELISA (Crystal Chem Inc., Downers Grove, IL, USA). Results were expressed as the concentration of insulin in the media relative to the concentration remaining in the pellet, to normalize for variability in the size of islets. Glucose-stimulated insulin release was determined by comparing the amount of insulin released from the pellet into the media at 16.7 mM glucose relative to 3 mM glucose.

### Statistical analysis

All statistical analyses were performed using SigmaStat (v.3.1, SPSS, Chicago, IL, USA). The results are expressed as mean±SEM. Data were checked for normality and equal variance and were tested using unpaired Student's *t*-tests (α = 0.05) at each age. Where data failed normality or equal variance test, data was reanalyzed using Mann-Whitney rank sum test.

## Results

### Oral glucose tolerance tests

**Figure 3:**
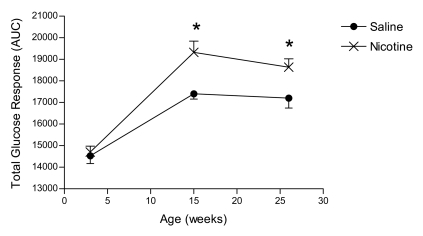
Oral glucose tolerance tests during postnatal development. Area under the curve (AUC) for the total glucose response to an oral glucose challenge at 4, 15 and 26 weeks of age in saline (closed circles) and nicotine-exposed (open circles) animals. All data are presented as mean±SEM. Values with an asterisk are significantly (p<0.05) different from saline controls.

At 4 weeks of age there was no effect of nicotine exposure (p>0.05) on the total glucose response (area under the curve; AUC) to the oral glucose load. By 15 weeks of age the nicotine-exposed animals had a higher total glucose response (AUC) relative to the saline controls (p<0.05), an effect which was also evident at 26 weeks of age (Figure 3).

### Mitochondrial structure

There was no difference in the number of mitochondria per beta cell area at any age (Figure 1A), but the mean individual mitochondrion area was significantly higher (p<0.05) following nicotine exposure at 15 and 26 weeks of age (Figure 1B) compared to saline controls. Furthermore, the proportion of mitochondria with either blebbing or merging with neighboring mitochondria dramatically increased in the nicotine-, but not saline-exposed animals with age (Figure 1C).

Structural abnormalities were also evident in mitochondria of nicotine-exposed offspring starting at weaning (postnatal day 21). At all ages, more than 75% of the mitochondria from saline-exposed offspring were classified as stage 1 (structurally intact; Figure 2A). In contrast, nicotine-exposed offspring had a significant decrease in the proportion of healthy, stage 1 mitochondria beginning at 3 weeks of age, followed by a continual decline with age, such that by 26 weeks only 31% of nicotine-exposed mitochondria were classified as stage 1 (Figure 2A). Coinciding with the loss of healthy stage 1 mitochondria was a significant 4.7-fold increase in the proportion of stage 2 mitochondria (visible swelling) at 3 weeks in the nicotine-exposed offspring (Figure 2B). Furthermore, by 15 weeks of age nearly 20% of the mitochondria in beta cells of nicotine-exposed offspring were classified as either stages 3, 4 or 5 and by 26 weeks this proportion had increased to 57% (Figure 2C–E). In contrast, at all ages examined less than 1% of the mitochondria from the saline control group were at stages 3, 4 and 5 combined (Figure 2C–E). It was not possible to perform statistics on the data presented in Figures 2C, D or E due to lack of variability in the saline treatment group replicates (nearly all 0%).

### Mitochondrial enzyme activity

**Figure 4:**
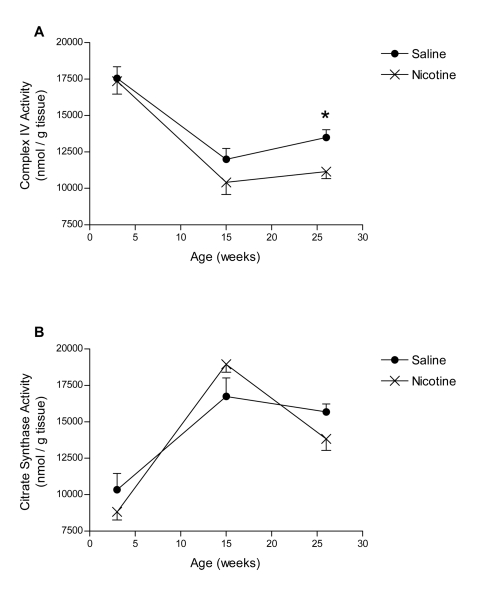
Mitochondrial enzyme activity during postnatal development. A) Complex IV enzyme activity (an indicator of mitochondrial electron transport chain function) and B) citrate synthase enzyme activity (an indicator of mitochondrial mass) in the pancreas of saline- and nicotine-exposed offspring at 3, 15 and 26 weeks of age. All data are presented as mean±SEM. Values with an asterisk are significantly (p<0.05) different from saline controls.

By 26 weeks, complex IV activity (a marker of mitochondrial function) was significantly reduced in the nicotine-exposed animals compared to saline controls (p<0.05; Figure 4A). There was no difference in citrate synthase activity (an indicator of mitochondrial mass) in the pancreas at any age examined (p>0.05; Figure 4B).

### Insulin granule characteristics

**Figure 5:**
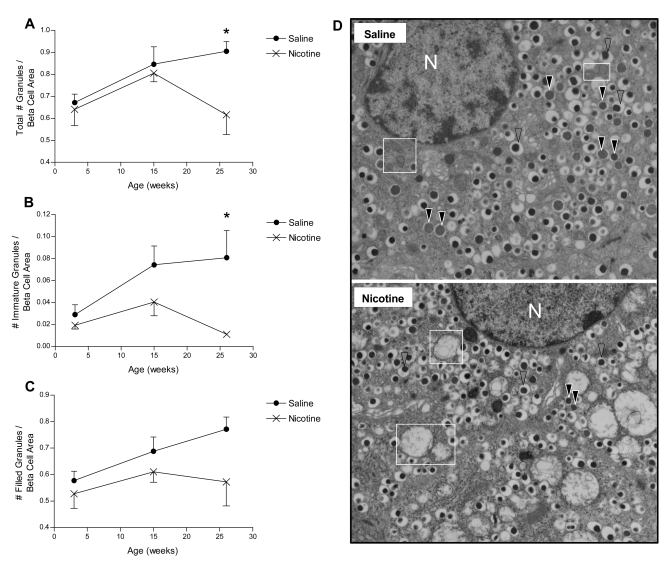
Insulin granule patterns during postnatal development. The number per beta cell area of: A) total insulin granules (filled, immature and empty); B) immature insulin secretory granules (containing pale-staining proinsulin); and C) filled insulin granules (containing dense-core mature insulin). D) Representative electron microscopy photographs of saline and nicotine-exposed beta cells at 26 weeks of age illustrate both the typical insulin granule patterns (immature insulin granules are indicated by solid black arrows and mature insulin granules by striped arrows), and mitochondrial structures (indicated by white boxes); N = nucleus. All data are presented as mean±SEM. Values with an asterisk are significantly (p<0.05) different from saline controls.

By 26 weeks of age, the nicotine-exposed animals had 32% fewer insulin granules in total (p<0.05; Figure 5A) and an 86% reduction in the number of immature granules per beta cell area (p<0.05; Figure 5B). The number of filled insulin granules per beta cell area was lower at all ages examined following nicotine exposure, but did not reach statistical significance (p>0.05; Figure 5C).

### Oxidative stress

**Figure 6:**
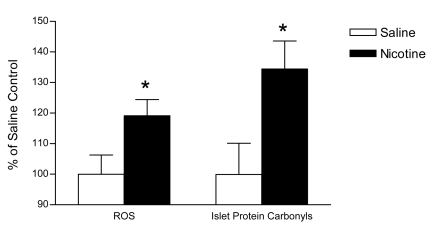
Oxidative stress at 26 weeks of age. Reactive oxygen species (ROS) production and the incidence of protein carbonyl groups (an indication of oxidative damage to protein) in islets isolated from the pancreas of saline- (white bar) and nicotine-exposed (black bar) offspring at 26 weeks of age. All data are expressed as a percentage of the average saline control value and are presented as the mean±SEM. Values with an asterisk are significantly (p<0.05) different from saline controls.

Islet ROS production in adult animals at 26 weeks of age was increased by approximately 20% following fetal and neonatal exposure to nicotine relative to saline (p<0.05; Figure 6). Furthermore, there was a 35% increase in the formation of protein carbonyl groups in the islets of nicotine-exposed offspring compared to saline controls (p<0.05; Figure 6).

### Glucose-stimulated insulin secretion

**Figure 7:**
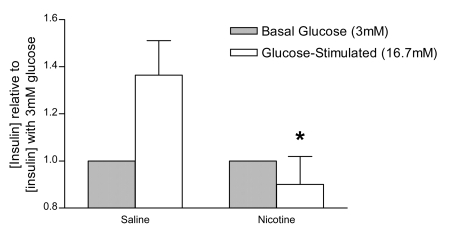
Glucose-stimulated insulin secretion at 26 weeks of age. Insulin release into the media was measured following glucose stimulation (16.7 mM; white bars) or basal glucose exposure (3.0 mM; striped bars) in saline- and nicotine-expose offspring at 26 weeks of age. All data are expressed as the insulin concentration normalized to the insulin concentration under basal glucose conditions (3.0 mM). Values with an asterisk indicate a significant difference in the stimulated/basal insulin release ratio for saline versus nicotine exposure (p<0.05). All data are presented as mean±SEM.

Fetal and neonatal exposure to nicotine resulted in impaired GSIS from pancreatic islets isolated from 26 week old animals (Figure 7).

## Discussion

Results from this study clearly demonstrate that fetal and neonatal nicotine exposure alters both mitochondrial structure and function postnatally. Mitochondrial structural abnormalities are observable prior to the onset of glucose intolerance and progressively worsen with age even though nicotine exposure is discontinued at weaning. Furthermore, as nicotine-exposed animals age, the observed mitochondrial defects appear to impact both mitochondrial function and beta cell function. These data raise concerns about the long term health consequences to the offspring following cigarette smoking or nicotine replacement therapy use during pregnancy and lactation.

The first observable mitochondrial alteration following developmental nicotine exposure was abnormal mitochondrial ultrastructure in the neonates at weaning (3 weeks of age). These early structural alterations in the nicotine-exposed offspring coincided with increases in both pancreatic oxidative stress [21] and mitochondrial-mediated beta cell apoptosis [22]. However, these changes in mitochondrial structure precede any observable alterations in glucose homeostasis. As the nicotine-exposed animals age, the proportion of beta cell mitochondria with severe structural abnormalities of the inner membrane (stages 3–5) and outer membrane (indicated by blebbing and/or merging) increased dramatically, despite discontinuation of nicotine exposure at weaning. These profound structural defects were not associated with any changes to the number of mitochondria within the beta cells, but were accompanied by a modest decline in mitochondrial enzyme activity, degranulation of beta cells, decreased beta cell function and impaired glucose tolerance (IGT). Therefore, we suggest that nicotine-induced mitochondrial damage has a significant role in the development of glucose intolerance in this animal model.

It is estimated that 98% of the energy for the beta cell is produced by mitochondrial oxidative metabolism [25]. Mitochondria are essential for both stages of glucose-stimulated insulin secretion from beta cells, including glucose entry and metabolism, as well as insulin exocytosis [25], [31]. In this study, fetal and neonatal nicotine exposure resulted in reduced complex IV enzyme activity at 26 weeks of age, an effect that was not associated with loss of mitochondrial number or mass. Since the respiratory chain enzymes are located within the inner membrane of the mitochondria, impairment of complex IV activity was expected given the observed deterioration of the inner membrane structural integrity in this animal model following perinatal nicotine exposure. Conversely, we did not detect a change in citrate synthase activity. However, citrate synthase is located within the mitochondrial matrix and therefore does not depend on mitochondrial membrane integrity. Furthermore, since citrate synthase is an indicator of mitochondrial mass, a decline in activity would only be expected if nicotine exposure resulted in a reduction in the number of mitochondria.

We propose that exposure of the beta cell mitochondria to reactive oxygen species (ROS) likely contributed to the loss of respiratory enzyme function and mitochondrial structural integrity in this animal model. ROS have been shown to inactivate the iron-sulfur centers of the electron transport chain complexes, thus causing defects in mitochondrial energy production [32]. In addition, when the function of one of the electron carrier complexes is impaired electrons are not shuttled properly through the electron transport chain (ETC) and are increasingly lost to molecular oxygen, resulting in increased ROS formation [19]. We hypothesize that in our animal model, this cycle was initiated during fetal and neonatal exposure to nicotine, a compound shown to have pro-oxidant properties *in vitro* and *in vivo* [33]–[35]. We have previously demonstrated that nicotine exposure during fetal and neonatal development leads to increased islet ROS production and oxidative damage at weaning [21]. We propose that this nicotine-induced increase in ROS likely triggered early, but undetectable damage to the ETC enzymes, thus initiating a feed-forward chain of progressive mitochondrial damage and additional ROS production. Furthermore, once dysglycemia has been established (by 15 weeks of age in the current study), chronic high glucose levels likely also contributed to the observed deterioration of pancreatic mitochondrial structure and function, as well as the loss of beta cell function. Chronic exposure to high glucose has previously been shown to induce mitochondrial-mediated beta cell apoptosis [36], as well as mitochondrial superoxide production and beta cell dysfunction in isolated islets [37]. As predicted, during adulthood nicotine-exposed offspring had elevated islet ROS production that was associated with increased formation of protein carbonyl groups in isolated islets, an indication that the redox balance has been disrupted in these cells. Therefore, in this animal model, perinatal nicotine exposure increases islet ROS production both at the end of lactation (i.e. during the nicotine exposure) [21] prior to the observable changes in ETC enzyme activity, and at 26 weeks of age when impaired complex IV activity and the most pronounced mitochondrial structural abnormalities were observed. We predict that the early mitochondrial structural alterations are likely initiated by nicotine-induced ROS, whereas the dramatic worsening of these defects between 15 and 26 weeks may be a consequence of chronic exposure to high glucose combined with ROS production by previously damaged mitochondrial ETC enzymes.

Although only a subtle reduction in respiratory enzyme activity was detected in the whole pancreas at 26 weeks following nicotine exposure, this may represent a more profound change in the beta cells, which comprise approximately 1% of the adult rodent pancreas [14]. Beta cells are known to be particularly susceptible to ROS damage since they have relatively low expression of antioxidant enzymes [38], [39]. We observed dramatic mitochondrial structural abnormalities by electron microscopy in the beta cells of nicotine-exposed offspring as early as 3 weeks of age. On the contrary, loss of respiratory enzyme activity measured in the whole pancreas was not detectable until 26 weeks. Therefore, it is possible that whole tissue measurements were simply not sensitive enough to detect differences between saline and nicotine exposure at the level of the beta cell in the younger animals. We propose that as the damage to mitochondrial protein accumulates with age, these changes become detectable at the whole tissue level.

Based on the numerous mitochondrial defects observed in this study (both structural and functional), it was expected that the nicotine-exposed offspring in this animal model would have altered beta cell function. As anticipated, developmental nicotine exposure resulted in altered insulin granule morphology and impaired GSIS compared to saline controls at 26 weeks of age. Electron microscopy (EM) analysis revealed a reduction in the total number of insulin granules within beta cells of nicotine-exposed offspring. Furthermore, there was a pronounced reduction (18-fold) in the number of pale, immature secretory granules by 26 weeks, suggesting that proinsulin biosynthesis may be impaired in this animal model and thus contribute to the loss of beta cell function. Indeed, proinsulin gene transcription has been previously shown to be crucial for maintaining proinsulin biosynthesis, retaining islet insulin stores, and ultimately regulating glucose homeostasis [40]. However, this finding of fewer immature insulin granules conflicts with EM studies in other animal models of dysglycemia, which have reported increased numbers of immature granules [41], [42]. These differences may be related to the profound alterations in mitochondrial structure observed in our model relative to previous studies. For example, in other models where the number of immature secretory granules was increased, no changes in mitochondrial structure were reported [41], [42]. In contrast, a significant proportion of the mitochondria in nicotine-exposed offspring were visibly swollen and vacuolated (stage 3–5) by 15 (20%) and 26 (56%) weeks of age. Another major difference between our animal model and the Zucker *fa/fa* rat model is that in Zucker *fa/fa* rats beta cells with a high proportion of immature granules had *increased* sensitivity to glucose, suggesting that the beta cells in this animal model are hyperactive [42]. In contrast, the nicotine-exposed rats in this animal model of dysglycemia exhibited a diminished ability to secrete insulin in response to a glucose stimulus. Similarly, transgenic mice with beta cell-specific mitochondrial defects have decreased GSIS (i.e. impaired beta cell function) [43], [44]. Therefore, the impaired GSIS observed in this study may be attributed to the inability of damaged mitochondria to: a) regulate proinsulin biosynthesis, and b) couple a glucose stimulus to insulin synthesis and exocytosis.

In conclusion, data from this study indicate that fetal and neonatal nicotine exposure adversely affects postnatal mitochondrial structure and function, which in turn leads to impaired beta cell function and dysglycemia in adult offspring. These data suggest a mechanism to explain, in part, the increased risk of type 2 diabetes in children born to women who smoked during pregnancy. This study also provides further support to the recent concerns about the safety of nicotine replacement therapy during pregnancy and lactation [45].
